# Employment Transitions and Mental Health in a Cohort of 45 Years and Older Australians

**DOI:** 10.3390/ijerph18179030

**Published:** 2021-08-27

**Authors:** Vikas Arya, Sandro Sperandei, Matthew J. Spittal, Andrew Page

**Affiliations:** 1 Translational Health Research Institute, Western Sydney University, Penrith, NSW 2571, Australia; s.sperandei@westernsydney.edu.au (S.S.); a.page@westernsydney.edu.au (A.P.); 2 Melbourne School of Population and Global Health, University of Melbourne, Parkville, VIC 3010, Australia; m.spittal@unimelb.edu.au

**Keywords:** mental health, employment transitions, psychological distress, unemployment

## Abstract

*Background:* This study investigated the associations between employment transitions and psychological distress among a cohort of 45 years and older Australians. *Methods:* This study was based on the 45 and Up Study, a large prospective cohort of participants aged 45 years and older (N = 267,153), followed up over the period 2006–2015. The risk of psychological distress was compared between various employment transitions categories by specifying an ordered logistic regression model adjusting for confounders. *Results:* Compared to participants who remained employed at baseline and follow-up, higher psychological distress was found among those who transitioned from being employed to unemployed (OR = 2.68, 95%CI 2.13–3.33) and to not being in the labour force or retired (OR = 2.21, 95%CI 1.85–2.62). Higher psychological distress was also evident among those who remained unemployed from baseline to follow-up (OR = 2.00, 95%CI 1.10–3.43), and those who transitioned from being retired to being unemployed (OR = 1.55, 95%CI 1.03–2.27). Conversely, lower psychological distress was found among those who transitioned from being unemployed to being employed (OR = 0.35, 95%CI 0.25–0.51). In general, lower psychological distress was found among ‘positive’ employment transitions (transitioning to being employed or retired). *Conclusions:* Policies focussing on re-employment in older age, as well as unemployment schemes, might be helpful in reducing psychological distress among middle- and old-age Australians.

## 1. Introduction

Evidence suggests that employment is associated with mental health benefits including reduced depression and anxiety symptoms, enhanced social support and increased self-esteem [1]. Conversely, unemployment has been associated with higher mortality and poorer mental health [2], including suicidal behaviour [3,4]. While there is debate about the direction of the causal link between employment and mental health (does unemployment ‘cause’ poor mental health or vice versa), available evidence generally supports the notion that employed individuals have better mental health compared to the unemployed [5,6].

Other forms of unemployment, such as retirement, are also associated with mental health consequences. For example, some studies indicate that compared to involuntary retirement (e.g., due to ill health, or to take care of a sick or disabled family member), voluntary retirement is associated with better mental health outcomes [7,8]. Additionally, risk of attempted suicide has been shown to be higher among those who retire involuntarily compared to those who retire voluntarily [9]. Retirement among older age-groups represents a significant life transition phase, which can be associated with financial insecurity, and social and psychological distress, especially for those retiring due to external pressures (e.g., redundancy or ill-health) [10,11]. Psychological distress is commonly used in epidemiological studies and population surveys as an indicator of the mental health of the population and can be defined as a state of emotional suffering experienced by an individual in response to the presence of stressors. Psychological distress is usually characterized by symptoms of anxiety and depression [12].

Studies generally investigate the association between employment status and mental health in cross sectional designs [13,14], or longitudinal designs that use current employment status as a determinant of subsequent mental health [15,16]. Few studies examine the association between transitions from being employed to unemployed (or vice versa) and mental health status [17]. In Australia, while studies have examined associations between retirement and employment status and mental health among older-age cohorts [14] as well as transitions between specific employment statuses (e.g., temporary employment vs. permanent employment) and mental health [18], no studies have investigated the associations between specific transitions in employment and retirement and mental health, during a period in the life course that may be associated with substantial social and economic change. Accordingly, this study investigates the association between employment transitions and mental health, based on a large prospective cohort study of participants aged 45 years and older.

## 2. Methods

### 2.1. Study Participants

Detailed information on the study participants has been published elsewhere [9]. Briefly, data from the Sax Institute′s 45 and Up Study (a large prospective cohort of individuals aged ≥45 years from New South Wales (NSW), Australia) were used for this study [19]. The Services Australia (formerly the Australian Government Department of Human Services) Medicare enrolment database was used to randomly sample prospective participants, which provides near complete coverage of the NSW population. Individuals residing in rural and remote areas and those aged >80 years were oversampled [19]. The baseline questionnaire, which was administered between February 2006 and December 2009, was completed by 267,153 individuals and included items relating to demographic and social characteristics (e.g., retirement and work, social connectedness, education), personal health behaviours (e.g., smoking, physical activity, dietary habits) and general health-related data (e.g., psychological distress, medication, disease and surgical history). All participants agreed to subsequent follow-up, as well as linkage of their health records with routinely collected health databases. Out of those invited, around 18% enrolled in the study (by filling out a mailed questionnaire with a consent form), representing roughly 11% of the total population of NSW aged ≥45 years. The cohort was prospectively followed up during 2006–2015 [19]. For the present study, only those participants who completed questionnaires at baseline and at the first follow-up (completed during 2012–2015) were included for analysis (N = 142,548).

Furthermore, participant data were deterministically linked to the Services Australia Medicare Benefits Schedule (MBS) database (which comprises all government-subsidised health and medical services). This record linkage was facilitated by the Sax Institute using a unique identifier provided by Services Australia. The MBS database information was available between the years 2004 and 2018. For the current study, the mean follow-up period was 122 ± 24.2 months, while the minimum follow-up period was 108 months (129 ± 9.8), after the baseline survey was conducted. In the MBS database, information on participants dated back to at least 19 months (51 ± 9.9), before the baseline survey was conducted. Institutional ethics approval for this project was provided by NSW Population & Health Services Research Ethics Committee (HREC/18/CIPHS/29); ACT Health Human Research Ethics Committee (2018.ETH.00174); ACT Calvary Public Hospital Bruce Human Research Ethics Committee (39–2018); and Western Sydney University Human Research Ethics Committee (RH12891). The conduct of the 45 and Up Study was approved by the University of New South Wales Human Research Ethics Committee (HREC).

### 2.2. Study Outcome

The outcome for the present study was current psychological distress at follow-up, which was assessed using The Kessler Psychological Distress Scale 10 (K10) [20]. The K10 is a widely used measure of psychological distress and involves asking participants a series of 10 questions about their emotional state in the most recent 4-week period. Each of the questions is answered on a five-level scale. Each question is scored from one (“none of the time”) to five (“all of the time”). Scores from the 10 questions are summed (with a minimum possible score of 10 and a maximum possible score of 50), with low scores indicating low levels of psychological distress and vice versa [20].

The summary psychological distress scores were categorised as none (score 10–20), mild (score 21–24), moderate (score 25–29), or severe (score ≥ 30) [20,21]. Transitions in psychological distress were defined for baseline to wave 1 follow-up, and were categorised as either a ‘decrease’ in psychological distress (from ‘moderate or severe’ to ‘none or mild’), an ‘increase’ in psychological distress (from ‘none or mild’ to ‘moderate or severe’), or ‘no change’ in psychological distress (where distress category remained unchanged at follow-up).

### 2.3. Exposure Variable

Participant employment status collected at baseline and at the first follow-up (during 2012–2015) was used to define the exposure variable. All the participants answered questions related to employment status including their ‘current work status’ and ‘reasons for retirement’. The ‘current work status’ required participants to highlight whether they were in full-time paid work, in part-time paid work, self-employed, doing unpaid work, completely retired/pensioner, studying, partially retired, look after home/family, disabled/sick, unemployed, or other. For the participants who reported being partially or completely retired, they were asked the age at which they retired and the reasons for retirement. These included: reached usual retirement age, lifestyle reasons, to care for family member/friend, ill health, made redundant, could not find a job, other.

The information on ‘employment status’ was used to classify all the participants into the following groups at each follow-up time point: (i) Employed (EM); (ii) Unemployed (UN); (iii) Retired (RT); or (iv) Not in the labour force and not retired (NILF). The employment category was defined as individuals in full-time or part-time employment, self-employed or unpaid work. The unemployment category was defined as those in the labour force and looking for employment. The retired category (two different categories in the original survey—voluntary and involuntary retirement) was defined as individuals who either voluntarily retired (due to reaching the retirement age, or other lifestyle reasons) or involuntarily retired (due to redundancy, care for a family member, or because they could not find a job). Preliminary analysis indicated that psychological distress scores for employment transitions did not generally differ among those who retired voluntarily and those who retired involuntarily and were regrouped under one ‘retired’ category. Finally, not in the labour force and not retired category was defined as those who could not work due to disability or illness, looking after home or family, full-time study, or ‘other’ reason.

Preliminary analysis indicated that psychological distress scores for employment transitions did not generally differ among those who retired voluntarily and those who retired involuntarily and were regrouped under one ‘retired’ category (RT). Categories of employment status were used to define each of the 16 possible transition combinations (Table 1). Over the study period (from baseline to follow-up), 51,109 participants maintained their ‘employment status’ as employed followed by retired (*n* = 32,040) and not being in the labour force or retired (*n* = 2364) (Table 1).

### 2.4. Potential Confounders

Potential confounders were also considered at follow-up including sex (male; female), age (45–54; 55–64; ≥65 years), marital status (Married, de facto, living with a partner; or Divorced, separated, widowed, single), migrant status (Australian-born; or Non-Australian born), and highest educational achievement (No school certificate or other qualification, School or intermediate certificate, High school or leaving certificate; Trade or apprenticeship, Certificate or diploma; University degree or higher). The Duke Social Support Index (DSSI) score [22], which was determined by adding across four specific questions related to social (religious groups or social clubs) and personal (friends and family) interactions, was also specified in the present study as a measure of social support. Mental health service use (yes; no) prior to participant enrolment in the study (2004–2006) was obtained from MBS data (Supplementary Table S1) and was used as a proxy measure for past mental health status.

### 2.5. Statistical Analysis

The descriptive characteristics of participants were presented as absolute and relative frequency for each employment status transition and other study factors. Associations between employment status transitions and psychological distress were modelled using ordered logistic regression, adjusting for the confounders noted above. Ordered logistic regression has a similar interpretation as logistic regression models. The model coefficients are the same for all outcome binary combinations, considering them as stages of the same process. The results were presented as odds ratios with 95% confidence intervals, with the group of participants who remained employed from baseline to follow up (EM–EM) used as the reference category. All analyses were performed using R, version 3.6.3 [23].

## 3. Results

A total of 16.1% (*n* = 22,989) of the participants were excluded from the analysis due to missing data in one of the variables, with the outcome variable (K10 score, baseline, or follow-up) the most frequently observed missing variable. The characteristics of the remaining 119,559 participants are presented in Table 2.

**Figure 1:**
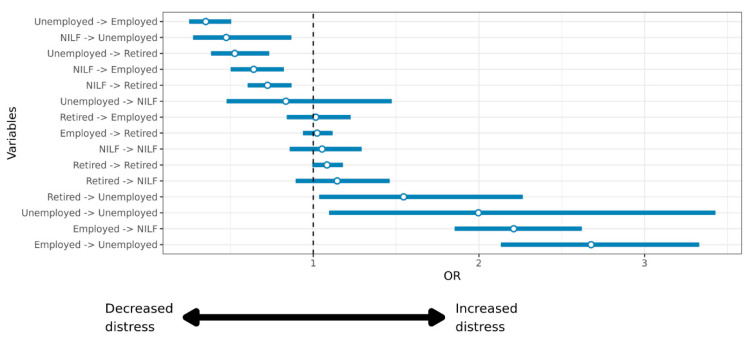
Relative risk of psychological distress by employment transitions over the follow-up period. NILF = Not in the labour force and not retired.

Compared to participants who remained employed at baseline and follow-up (EM–EM), psychological distress increased among those who transitioned from being employed (EM) to being unemployed (UN) (OR = 2.68, 95%CI 2.13–3.33) and to not being in the labour force or retired (NILF) (OR = 2.21, 95%CI 1.85–2.62). An increase in psychological distress was also evident among those who remained unemployed from baseline to follow-up (UN–UN) (OR = 2.00, 95%CI 1.10–3.43), those who transitioned from being retired (RT) to being unemployed (UN) (OR = 1.55, 95%CI 1.03–2.27) and to a lesser extent, those who transitioned from being retired (RT) to being not in the labour force or retired (NILF) (OR = 1.14, 95%CI 0.89–1.46) (Table 2; Figure 1).

Conversely, compared to participants who remained employed from baseline to follow-up (EM–EM), odds of decrease in psychological distress levels were higher among those who transitioned from being unemployed (UN) to being employed (EM) (OR = 0.35, 95%CI 0.25–0.51), those who transitioned from not being in the labour force or retired (NILF) to being unemployed (UN) (OR = 0.47, 95%CI 0.27–0.87), those who transitioned from being unemployed (UN) to being retired (RT) (OR = 0.53, 95%CI 0.38–0.73), those who transitioned from being not in the labour force or retired (NILF) to being employed (EM) (OR = 0.64, 95%CI 0.50–0.82), and those who transitioned from not being in the labour force or retired (NILF) to being retired (RT) (OR = 0.72, 95%CI 0.60–0.87) (Table 2; Figure 1).

Prior use of mental health services was also associated with an increased risk of psychological distress (OR = 1.15, 95%CI 1.08–1.22), while increased social support (as measured by the Duke Social Support Index) was associated with decreased risk of psychological distress (OR = 0.93, 95%CI 0.92–0.95) (Table 2).

## 4. Discussion

This study investigated associations between employment and retirement transitions and psychological distress among a cohort of older aged Australians (aged ≥ 45 years) over the period 2006–2015. Findings indicated that participants who stayed, or transitioned to being, employed or retired had lower risk of experiencing psychological distress compared to those who stayed, or transitioned to being, unemployed or not in the labour force, during the study period. The study also found prior use of mental health services to be associated with higher psychological distress and greater social support to be associated a lower risk of experiencing psychological distress.

The present study has some limitations. Firstly, the two surveys (baseline and follow-up) were administered 6–9 years apart. Therefore, we could not ascertain whether the employment transitions that our study captured were the only transitions that occurred over the study period, which could have had an impact on reported psychological distress levels. Secondly, ‘employment status’ and psychological distress were measured at the same time, which makes it difficult to ascertain whether psychological distress occurred prior or subsequent to the reported ‘employment status’. However, this was not the case for specifying employment transitions, where baseline employment status occurred prior to the measurement of psychological distress. Thirdly, the study could not ascertain the duration of participant ‘employment status’. For example, if a participant had only been unemployed for a few days, they might have reported less psychological distress compared to a participant who had been unemployed for months or years. Additionally, the 45 and Up Study had a response rate of 18%, representing a source of potential selection bias. For example, individuals with higher psychological distress may have been less likely to have participated in the study (either at baseline or at follow-up), resulting in potential underestimation of the association between employment transitions and psychological distress. However, comparisons between the 45 and Up Study and representative health surveys have indicated that selection bias does not substantially affect relative differences across a range of exposures and outcomes [24].

Despite these limitations, the present study makes an important contribution towards rarely studied associations between employment transitions and mental health, especially in the Australian context. Among all the employment transitions investigated, this study found that the highest risk of increased psychological distress was among individuals transitioning from being employed to being unemployed, and from being employed to not being in the labour force and not retired. This is consistent with previous evidence that unemployed individuals have higher psychological distress compared to the employed [25]. While being unemployed and not being in the labour force and not retired are two separate employment states, they are both characterised by not being able to work, either due to being unable to find employment, or being unable to look for employment. The negative impact of unemployment on mental health can be explained through perhaps two main mechanisms. Firstly, unemployment usually results in loss of income, which can adversely impact an individual’s ability to afford necessities such as housing, health care and food. All of these factors are associated with poor mental health [26]. Secondly, unemployment generally produces social consequences including loss of self-esteem, time structure and work-relationships, all of which might have negative mental health consequences [27,28].

The current study also suggests that transitioning from not being in the labour force (and not retired) to being employed or being unemployed is associated with lower risk of psychological distress. Lower risk of psychological distress from transitioning to being unemployed is an interesting finding, given the negative impacts of unemployment on mental health discussed above. However, this transition is indicative of moving to a state of being able to look for work as part of the labour force, perhaps reflecting a change in the circumstances associated with not being in the labour force and subsequently, lower psychological distress. However, this is likely dependent on the time elapsed between transitions, with any beneficial psychological impacts associated with being able to search for work likely to attenuate with longer term unemployment. 

Some evidence suggests that retirement has a positive effect of mental health [29], however, this might be limited to voluntary retirement [7,8]. The present study found evidence of lower risk of psychological distress among participants transitioning to retirement from being unemployed or not being in the labour force whereas higher psychological distress was evident among participants transitioning from retirement to unemployment. These results were consistent among the voluntary and involuntary retirement categories, further emphasizing the increased risk of psychological distress associated with unemployment. Some of the reasons for benefits of retirement, especially voluntary retirement, on mental health may include relinquishing work-related demands and stress, having more time to pursue activities of interest, having more time to exercise, and financial security for some [30].

Adjustment for sex, education, marital status, or immigration background did not substantially attenuate associations between employment transitions and risk of psychological distress. Limited evidence on various employment transitions and mental health highlights that transitioning from paid employment to unemployment is associated with increased psychological distress for both males and females and among different age-groups [17]. This might suggest that the impact of adverse employment transitions on psychological distress is important irrespective of other socio-cultural determinants, however, more research is warranted to further investigate these associations.

## 5. Conclusions

Previous studies have predominantly focused on employment status and mental health and have found that unemployment is generally associated with worse mental health outcomes compared to employment. The present study is one of the few studies that focuses on employment transitions and psychological distress. The findings suggest that transitioning to unemployment or to not being in the labour force or retired, especially from being employed, is associated with greater risk of psychological distress compared to staying (or transitioning to) employed or retired. Furthermore, transitioning to employment, especially from unemployment, is associated with decreased psychological distress. While more research is warranted to further explore the associations between employment transitions and mental health in Australia, policies and initiatives focusing on re-employment in older age and unemployment schemes might have mental health benefits for middle and older age Australians.

## Figures and Tables

**Table 1:** Number of participants by employment transition from baseline to follow-up.

			*Follow-Up*		
		EM	RT	UN	NILF
	EM	51,109 (69.4)	18,693 (25.4)	1307 (1.8)	2536 (3.4)
***Baseline***	RT	2773 (7.5)	32,040 (86.7)	552 (1.5)	1605 (4.3)
	UN	538 (29)	784 (42.3)	232 (12.5)	298 (16.1)
	NI	1443 (20.3)	3047 (43)	238 (3.4)	2364 (33.3)

EM = Employed; RT = Retired, UN = Unemployed; NILF = Not in the labour force and not retired.

**Table 2:** Relative risk of psychological distress at follow-up by employment transitions and other study factors (6–9 years of follow-up, NSW 45 and Up Study, 2006–2015).

	Psychological Distress over the Follow-Up Period				
	Decrease	No Change	Increase	OR (95%CI) (b)	OR (95%CI) (c)
*Employment transitions (a)*					
**From being employed at baseline:**					
Employed–Employed	977 (38.7)	49,099 (43)	1033 (36.1)	1.00	1.00
Employed–Retired	275 (10.9)	18,113 (15.9)	305 (10.7)	1.01 (0.93–1.09)	1.02 (0.94–1.12)
Employed–Unemployed	40 (1.6)	1160 (1)	107 (3.7)	2.78 (2.21–3.44)	2.68 (2.13–3.33)
Employed–NILF	89 (3.5)	2259 (2)	188 (6.6)	2.26 (1.89–2.68)	2.21 (1.85–2.62)
**From being retired at baseline:**					
Retired–Employed	37 (1.5)	2697 (2.4)	39 (1.4)	0.99 (0.82–1.19)	1.01 (0.84–1.23)
Retired–Retired	504 (20)	30,882 (27)	654 (22.8)	1.09 (1.01–1.16)	1.08 (0.99–1.18)
Retired–Unemployed	14 (0.6)	512 (0.4)	26 (0.9)	1.60 (1.07–2.33)	1.55 (1.03–2.27)
Retired–NILF	70 (2.8)	1451 (1.3)	84 (2.9)	1.20 (0.93–1.52)	1.14 (0.89–1.46)
**From being unemployed at baseline:**					
Unemployed–Employed	40 (1.6)	483 (0.4)	15 (0.5)	0.37 (0.26–0.53)	0.35 (0.25–0.51)
Unemployed–Retired	44 (1.7)	716 (0.6)	24 (0.8)	0.55 (0.40–0.76)	0.53 (0.38–0.73)
Unemployed–Unemployed	15 (0.6)	194 (0.2)	23 (0.8)	2.12 (1.16–3.64)	2.00 (1.10–3.43)
Unemployed–NILF	26 (1)	247 (0.2)	25 (0.9)	0.90 (0.51–1.58)	0.83 (0.48–1.47)
**From being NILF at baseline:**					
NILF–Employed	68 (2.7)	1332 (1.2)	43 (1.5)	0.66 (0.51–0.84)	0.64 (0.50–0.82)
NILF–Retired	153 (6.1)	2775 (2.4)	119 (4.2)	0.75 (0.62–0.89)	0.72 (0.60–0.87)
NILF–Unemployed	21 (0.8)	203 (0.2)	14 (0.5)	0.50 (0.28–0.91)	0.47 (0.27–0.87)
NILF–NILF	152 (6)	2048 (1.8)	164 (5.7)	1.10 (0.89–1.35)	1.05 (0.86–1.29)
*Sex*					
Female	1540 (61)	62,246 (54.5)	1719 (60)	1.00	1.00
Male	985 (39)	51,925 (45.5)	1144 (40)	1.00 (0.95–1.06)	0.98 (0.93–1.04)
*Age group*					
Up to 54	411 (16.3)	12,129 (10.6)	424 (14.8)	1.00	1.00
55–64	1176 (46.6)	45,609 (39.9)	1318 (46)	1.05 (0.95–1.14)	1.06 (0.96–1.16)
65 and above	938 (37.1)	56,433 (49.4)	1121 (39.2)	1.05 (0.95–1.15)	1.09 (0.98–1.21)
*Educational achievement*					
University degree or higher	574 (22.7)	35,532 (31.1)	649 (22.7)	1.00	1.00
Trade, Certificate or Diploma	868 (34.4)	38,093 (33.4)	960 (33.5)	1.01 (0.94–1.07)	1.00 (0.93–1.07)
High school completion or lower	1083 (42.9)	40,546 (35.5)	1254 (43.8)	1.05 (0.70–2.62)	1.03 (0.96–1.10)
*Migrant status*					
Australian-born	2012 (79.7)	89,623 (78.5)	2216 (77.4)	1.00	1.00
Not Australian-born	513 (20.3)	24,548 (21.5)	647 (22.6)	1.07 (1.00–1.14)	1.05 (0.98–1.12)
*Previous mental health service use (d)*					
No	1085 (43)	86,267 (75.6)	1195 (41.7)	1.00	1.00
Yes	1440 (57)	27,904 (24.4)	1668 (58.3)	1.15 (1.08–1.22)	1.15 (1.08–1.22)
*Marital status*					
Married/de facto	1603 (63.5)	87,217 (76.4)	1880 (65.7)	1.00	1.00
Unmarried/widowed	922 (36.5)	26,954 (23.6)	983 (34.3)	0.98 (0.91–1.04)	0.96 (0.90–1.02)
DSSI (e)	8.2 (1.9)	8.7 (1.7)	7.9 (1.9)	0.94 (0.92–0.95)	0.93 (0.92–0.95)

(a) Transitions presented as “From baseline” “To follow-up”; NILF = Not in the labour force and not retired; (b) Unadjusted; (c) Adjusted for sex, age-group, educational achievement, migrant status, previous mental health service use, marital status, DSSI; (d) Based on MBS mental health service items (see Supplementary Table S1); (e) DSSI: Duke Social Support Index.

## Data Availability

Data from this study cannot be shared due to Data Custodian agreements relating to the access and use of the linked 45 and Up Study data held by the Sax Institute. Code can be provided by the authors on request.

## Supplementary Materials

The following are available online at https://www.mdpi.com/article/10.3390/ijerph18179030/s1, Table S1: Summary of Medicare Benefits Schedule (MBS) mental health service items.

## References

[1] Modini M., Joyce S., Mykletun A., Christensen H., Bryant R.A., Mitchell P.B., Harvey S.B. (2016). The mental health benefits of employment: Results of a systematic meta-review. Australas. Psychiatry.

[2] Kasl S.V., Jones B.A. (2000). The impact of job loss and retirement on health. Soc. Epidemiol..

[3] Milner A., Page A., LaMontagne A.D. (2013). Duration of unemployment and suicide in Australia over the period 1985–2006: An ecological investigation by sex and age during rising versus declining national unemployment rates. J. Epidemiol. Community Health.

[4] Milner A., Page A., LaMontagne A.D. (2013). Long-term unemployment and suicide: A systematic review and meta-analysis. PLoS ONE.

[5] Murphy G.C., Athanasou J.A. (1999). The effect of unemployment on mental health. J. Occup. Organ. Psychol..

[6] Hergenrather K.C., Zeglin R.J., McGuire-Kuletz M., Rhodes S.D. (2015). Employment as a social determinant of health: A review of longitudinal studies exploring the relationship between employment status and mental health. Rehabil. Res. Policy Educ..

[7] Rhee M.K., Mor Barak M.E., Gallo W.T. (2016). Mechanisms of the effect of involuntary retirement on older adults’ self-rated health and mental health. J. Gerontol. Soc. Work..

[8] Mosca I., Barrett A. (2016). The impact of voluntary and involuntary retirement on mental health: Evidence from older Irish adults. J. Ment. Health Policy Econ..

[9] Page A., Sperandei S., Spittal M.J., Milner A., Pirkis J. (2020). The impact of transitions from employment to retirement on suicidal behaviour among older aged Australians. Soc. Psychiatry Psychiatr. Epidemiol..

[10] Alavinia S.M., Burdorf A. (2008). Unemployment and retirement and ill-health: A cross-sectional analysis across European countries. Int. Arch. Occup. Environ. Health.

[11] Van Solinge H., Henkens K. (2008). Adjustment to and satisfaction with retirement: Two of a kind?. Psychol. Aging.

[12] Drapeau A., Marchand A., Beaulieu-Prévost D. (2012). Epidemiology of psychological distress. Ment. Illn. Underst. Predict. Control.

[13] Schneider B., Grebner K., Schnabel A., Hampel H., Georgi K., Seidler A. (2011). Impact of employment status and work-related factors on risk of completed suicide: A case–control psychological autopsy study. Psychiatry Res..

[14] Vo K., Forder P.M., Tavener M., Rodgers B., Banks E., Bauman A., Byles J.E. (2015). Retirement, age, gender and mental health: Findings from the 45 and Up Study. Aging Ment. Health.

[15] Fergusson D.M., Horwood L.J., Woodward L.J. (2001). Unemployment and psychosocial adjustment in young adults: Causation or selection?. Soc. Sci. Med..

[16] Butterworth P., Leach L.S., Strazdins L., Olesen S.C., Rodgers B., Broom D. (2011). The psychosocial quality of work determines whether employment has benefits for mental health: Results from a longitudinal national household panel survey. Occup. Environ. Med..

[17] Thomas C., Benzeval M., Stansfeld S.A. (2005). Employment transitions and mental health: An analysis from the British household panel survey. J. Epidemiol. Community Health.

[18] LaMontagne A.D., Milner A., Krnjacki L., Kavanagh A.M., Blakely T.A., Bentley R. (2014). Employment arrangements and mental health in a cohort of working Australians: Are transitions from permanent to temporary employment associated with changes in mental health?. Am. J. Epidemiol..

[19] 45 and Up Study Collaborators (2008). Cohort profile: The 45 and up study. Int. J. Epidemiol..

[20] Kessler R.C., Andrews G., Colpe L.J., Hiripi E., Mroczek D.K., Normand S.L., Walters E., Zaslavsky A.M. (2002). Short screening scales to monitor population prevalences and trends in non-specific psychological distress. Psychol. Med..

[21] Andrews G., Slade T. (2001). Interpreting scores on the Kessler psychological distress scale (K10). Aust. N. Z. J. Public Health.

[22] Koenig H.G., Westlund R.E., George L.K., Hughes D.C., Blazer D.G., Hybels C. (1993). Abbreviating the Duke Social Support Index for use in chronically ill elderly individuals. Psychosomatics.

[23] R Core Team (2020). R: A Language and Environment for Statistical Computing.

[24] Mealing N.M., Banks E., Jorm L.R., Steel D.G., Clements M.S., Rogers K.D. (2010). Investigation of relative risk estimates from studies of the same population with contrasting response rates and designs. BMC Med Res. Methodol..

[25] Paul K.I., Moser K. (2009). Unemployment impairs mental health: Meta-analyses. J. Vocat. Behav..

[26] Lund C. (2012). Poverty and mental health: A review of practice and policies. Neuropsychiatry.

[27] Warr P. (1987). Work, Unemployment, and Mental Health.

[28] Goldsmith A.H., Veum J.R., William D. (1996). The impact of labor force history on self-esteem and its component parts, anxiety, alienation and depression. J. Econ. Psychol..

[29] Van der Heide I., van Rijn R.M., Robroek S.J., Burdorf A., Proper K.I. (2013). Is retirement good for your health? A systematic review of longitudinal studies. BMC Public Health.

[30] Oksanen T., Vahtera J., Westerlund H., Pentti J., Sjösten N., Virtanen M., Kawachi I., Kivimäki M. (2011). Is retirement beneficial for mental health? Antidepressant use before and after retirement. Epidemiology.

