# Supplementary material for: Employment Transitions and Mental Health in a Cohort of 45 Years and Older Australians
Source: Int J Environ Res Public Health. 2021 Aug 27;18(17):9030. doi: 10.3390/ijerph18179030 (PMC8430720; doi:10.3390/ijerph18179030)
Supplement: Supplementary file 1 [file ijerph-18-09030-s001.zip › ijerph-1351698-supplementary.pdf]

Supplementary Table S1: Summary of Medicare Benefit Schedule (MBS) mental health service items

Group code	Group	Item Numbers
A8	Consultant Psychiatrist Attendance	288, 289, 291, 293, 296, 297, 299, 300, 302, 304, 306, 308, 310, 312, 314, 316, 318, 319, 320, 322, 324, 326, 328, 330, 332, 334, 336, 338, 342, 344, 346, 348, 350, 352, 353, 355, 356, 357, 358, 359, 361, 364, 366, 367, 369, 370
A15	GP Management Plans, Team Care Arrangements, Multidisciplinary Care Plans	855, 857, 858, 861, 864, 866
T1	Miscellaneous Therapeutic Procedures	14224
M6	Psychological Therapy Services	80000, 80005, 80010, 80015, 80020
A6	Group Therapy	170, 171, 172
A18	GP attendance associated with PIP incentive payment	2574, 2575, 2577, 2578
A20	GP mental health care	2700, 2701, 2702
A19	Other non-referred attendance associated with PIP	2704, 2705, 2707, 2708
A20	GP mental health treatment	2710, 2712, 2713, 2715, 2717, 2719, 2721, 2723, 2725, 2727
T10	Relative value guide for Anesthesia	20104
M3	Allied Health Services	10956, 10968
M7	Focussed Psychological Strategies	80100, 80105, 80110, 80115, 80120, 80125, 80130, 80135, 80140, 80145, 80150, 80155, 80160, 80165, 80170
M11	Allied Health Services For Indigenous Australians	81325, 81355
M10	Autism, Pervasive Developmental Disorder And Disability Services	82000, 82015
